# E-cigarette side effects in otolaryngology: unveiling the vape mirage

**DOI:** 10.1038/s41432-023-00941-0

**Published:** 2023-10-20

**Authors:** Gabriele Baniulyte, Kamran Ali

**Affiliations:** 1 Oral and Maxillofacial Department, Royal Devon and Exeter Hospital, Barrack Road, Exeter, EX2 5DW UK; 2 QU Health, College of Dental Medicine, Oral Surgery, Qatar University, Doha, 2713 Qatar

**Keywords:** Dentistry, Health care

## Abstract

**Data sources:**

The following databases were searched for publications up to May 2020: Web of Science, EMBASE, CENTRAL, Medline and CINAHL. Additionally, previously published reviews were hand searched.

**Study selection:**

Clinical studies conducted in English language were considered, encompassing cohorts of more than four vaping individuals who have encountered inadvertent side effects. Both adult and paediatric populations were included. In vitro, animal studies and systematic or literature reviews were excluded from the analysis.

**Data extraction and synthesis:**

Two independent reviewers screened 1125 studies following deduplication. Two-hundred and eight full-text studies were assessed for eligibility.

**Results:**

Thirty-two studies met the inclusion criteria. Diverse study designs were included, comprising of cross-sectional, randomised controlled trials, case-control studies, cohort studies, case series investigations and non-randomised trials. Of note, four studies focused on paediatric patients. Most reported side effects were cough, throat and mouth irritation and intra-oral lesions.

**Conclusions:**

While the direct side effects of e-cigarettes are well-documented, the long-term effects remain uncertain.

## A Commentary on

**Amanian A, Phulka J, Hu A C**.

Unintended side effects of electronic cigarettes in otolaryngology: a scoping review. *Otolaryngol Head Neck Surg* 2023; 10.1177/01945998211069502.

**GRADE Rating:** 

## Commentary

The rapid evolution of electronic cigarette (e-cigarette) technology and the continuous influx of new products have pushed us into the future where the boundaries of nicotine consumption are constantly redrawn. This is sparking debate about addiction, health, and the future of tobacco control. Striking a balance between protecting public health, especially among youth, and providing adult smokers with potentially less harmful alternatives remains a challenge for policymakers worldwide^1^.

This review aimed to explore the short-term side effects of e-cigarette use in otolaryngology setting. Joanna Briggs Institute methodology was followed for reporting and the quality of studies was assessed using Oxford levels of evidence. Five databases were searched from inception to May 2020, including hand-searching previous reviews, by two independent examiners. Inclusion criteria encompassed studies conducted in the English language, involving cohorts of more than four participants who engage in vaping and report otolaryngology-related side effects. Both adult and paediatric populations were included while excluding systematic or literature reviews, animal and *in vitro* studies, as well as studies exclusively focused on lower airway side effects.

This review incorporated a total of 32 studies. Among these, thirteen adopted a cross-sectional design, nine were randomised controlled trials, five were cohort studies, three followed case-control methodologies, one was a case series and one a non-randomised trial. Sample sizes across these studies ranged from 8 to 65,528 participants. Among the included studies, four specifically investigated side effects within the paediatric population. Notably, nicotine emerged as the predominant substance of choice in vaping, with many users concurrently reporting cigarette or tobacco use alongside e-cigarettes. A striking finding from one study was that nicotine inhalators were associated with a higher incidence of mouth and throat irritation when compared to nicotine vapour. However, a randomised controlled trial between healthy individuals and asthmatic patients using a nicotine-free and flavour-free vaping device yielded inconclusive results, showing no conclusive differences in the side effects.

Among the studies published in otolaryngology, seventeen reported the occurrence of cough alongside symptoms such as sore throat, dry mouth, or throat, as well as mouth or throat irritation. In most instances, these symptoms resolved within several months; however, one study documented the persistence of these symptoms even after 24 months. Additionally, a separate study reported eight patients who required hospitalisation due to acute respiratory failure, with the majority having used marijuana-based vaping devices. Among the otology studies, eight highlighted vestibular dysfunction, while one study indicated hearing loss attributed to e-cigarette use. Within the realm of head and neck sub-specialties, four studies identified an elevated incidence of oral mucosal lesions, including hyperplastic candidiasis, hairy tongue, and nicotine stomatitis. In laryngology, one study associated e-cigarette use with a harsh voice quality, and in rhinology, one study reported sinusitis, while two paediatric studies reported allergic rhinitis.

While this review was well designed and conducted, several limitations should be mentioned. Initially, the intention was to conduct a systematic review; however, the inherent heterogeneity and the absence of suitable high-level studies during the initial search compelled a pivot towards a scoping review methodology. This shift, while practical, may warrant a nuanced interpretation of the review’s scope and depth.

Another limitation arises from the exclusion of grey literature, which potentially overlooks valuable insights and data beyond traditional academic sources. Furthermore, some of the studies included remarkably small sample sizes, as low as 8 participants, limiting the generalisability of findings. Additionally, the presence of a study investigating side effects related to marijuana-based products rather than nicotine introduces a deviation from the primary focus of this review. Moreover, the vast diversity among e-cigarette products, especially those containing nicotine versus those without, could lead to varying health effects and outcomes. Consequently, the broad search strategy which included any e-cigarettes, with and without nicotine or marijuana, may have resulted in the aggregation of studies with different characteristics. Lastly, the incorporation of both adult and paediatric populations, while broadening the review’s applicability, should be approached with caution. The inherent differences in physiology and behaviours between these age groups may introduce significant heterogeneity, potentially impacting the ability to draw meaningful conclusions for either group. The above-mentioned limitations underscore the importance of considering the context and constraints within which this scoping review was conducted.

While e-cigarettes are purportedly designed to facilitate smoking cessation, their marketing and flavouring tactics have faced criticism for potentially enticing a population already prone to addiction, notably adolescents, in the absence of robust long-term evidence to substantiate their safety claims^2,3^. Furthermore, the prevalent use of e-cigarettes in public spaces appears to normalise smoking behaviours, especially among youth who increasingly perceive vaping as a socially acceptable alternative^4^. This normalisation threatens to undermine the painstaking efforts aimed at reducing smoking rates and shielding nonsmokers from harmful second-hand exposure. E-cigarette aerosol is a complex mixture comprising nicotine, flavourings, and various additives. Even in its capacity as second-hand aerosol, some of these chemicals may pose risks, particularly for vulnerable populations like children and individuals with pre-existing health conditions^5^. It is worth noting that e-cigarette companies are capitalising on the lack of long-term data to assert the safety of their products. These concerns underscore the need for continuing research, vigilant regulation, and proactive public health initiatives to safeguard individuals and communities.

Practice points

- Highlights the short-term side effects of using e-cigarettes include cough, throat and mouth irritation and intra-oral lesions.
- Emphasises the need to inform public about the potential consequences of second-hand exposure to e-cigarette vapour.
